# Refining Ovarian Cancer Test accuracy Scores (ROCkeTS): protocol for a prospective longitudinal test accuracy study to validate new risk scores in women with symptoms of suspected ovarian cancer

**DOI:** 10.1136/bmjopen-2015-010333

**Published:** 2016-08-09

**Authors:** Sudha Sundar, Caroline Rick, Francis Dowling, Pui Au, Kym Snell, Nirmala Rai, Rita Champaneria, Hilary Stobart, Richard Neal, Clare Davenport, Susan Mallett, Andrew Sutton, Sean Kehoe, Dirk Timmerman, Tom Bourne, Ben Van Calster, Aleksandra Gentry-Maharaj, Usha Menon, Jon Deeks

**Affiliations:** 1 Institute of Cancer and Genomic Sciences, University of Birmingham, Birmingham, UK; 2 Pan Birmingham Gynaecological Cancer Centre, City Hospital, Birmingham, UK; 3 Birmingham Clinical Trials Unit, University of Birmingham, Birmingham, UK; 4 Diagnostic Test Accuracy Group, University of Birmingham, Birmingham, UK; 5 Patient Representative, Birmingham, UK; 6 Primary Care, Bangor University, North Wales, UK; 7 Health Economics, University of Birmingham, Birmingham, UK; 8 Department of Development and Regeneration, KU Leuven, Leuven, Belgium; 9 Department of Obstetrics and Gynaecology, KU Leuven, Leuven, Belgium; 10 Queen Charlotte's and Chelsea Hospital, Imperial College, London, UK; 11 Department of Women's Cancer, Gynaecological Cancer Research Centre, Institute for Women's Health, UCL, London, UK

**Keywords:** GYNAECOLOGY

## Abstract

**Introduction:**

Ovarian cancer (OC) is associated with non-specific symptoms such as bloating, making accurate diagnosis challenging: only 1 in 3 women with OC presents through primary care referral. National Institute for Health and Care Excellence guidelines recommends sequential testing with CA125 and routine ultrasound in primary care. However, these diagnostic tests have limited sensitivity or specificity. Improving accurate triage in women with vague symptoms is likely to improve mortality by streamlining referral and care pathways. The Refining Ovarian Cancer Test Accuracy Scores (ROCkeTS; HTA 13/13/01) project will derive and validate new tests/risk prediction models that estimate the probability of having OC in women with symptoms. This protocol refers to the prospective study only (phase III).

**Methods and analysis:**

ROCkeTS comprises four parallel phases. The full ROCkeTS protocol can be found at http://www.birmingham.ac.uk/ROCKETS. Phase III is a prospective test accuracy study. The study will recruit 2450 patients from 15 UK sites. Recruited patients complete symptom and anxiety questionnaires, donate a serum sample and undergo ultrasound scored as per International Ovarian Tumour Analysis (IOTA) criteria. Recruitment is at rapid access clinics, emergency departments and elective clinics. Models to be evaluated include those based on ultrasound derived by the IOTA group and novel models derived from analysis of existing data sets. Estimates of sensitivity, specificity, c-statistic (area under receiver operating curve), positive predictive value and negative predictive value of diagnostic tests are evaluated and a calibration plot for models will be presented. ROCkeTS has received ethical approval from the NHS West Midlands REC (14/WM/1241) and is registered on the controlled trials website (ISRCTN17160843) and the National Institute of Health Research Cancer and Reproductive Health portfolios.

**Strengths and limitations of this study**

- ROCkeTS conforms to the PROBE design for biomarker evaluation and STARD criteria for test accuracy studies.
- Stringent efforts to collect outcome data to prevent ascertainment bias.
- Inbuilt ultrasound quality control.
- Recruitment is at secondary care, so the population will be less heterogeneous than that is seen in primary care.

## Introduction

Ovarian cancer (OC) is the seventh most common cancer in women worldwide, with 239 000 new cases diagnosed in 2012.^1^ In the UK, OC has an annual incidence of 7116 women and causes 4271 deaths; the lifetime risk of developing OC is 1 in 54.^2^ Seventy per cent of patients will present at an advanced stage (stages III/IV). The International Cancer Benchmarking Project showed that the reason that OC survival in the UK is significantly lower than other western countries seems to be related to a lower proportion of patients receiving treatment and surviving the first year after cancer diagnosis and is likely due to a delay in diagnosis.^3^ Five-year survival rates are 43% overall but over 90% for early-stage tumours.^4^ High-grade serous is the most common histotype (80%). Worryingly, long-term survival from OC has remained static over the past decades at 30%.^2^

OC was previously considered a ‘silent killer’; it is now recognised that patients with OC suffer from a number of non-specific symptoms. These include abdominal bloating, distension, feeling full quickly and/or loss of appetite, pelvic/abdominal pain, increased urinary urgency and/or frequency, unexplained weight loss, fatigue or changes in bowel habit. These symptoms are very common.^5^ ^6^ Interrogation of UK General Practice databases suggest that on average one in two women between the ages of 45 and 74 presents once a year to her general practitioner (GP)/primary care doctor with these symptoms. Abdominal bloating alone^5^ ^6^ is documented in 16–30% of women presenting to GPs.^7^ Diagnostic challenges are considerable given (1) the low incidence of OC (a GP sees a woman with OC once in 3–5 years) and (2) the low positive predictive value (PPV) of symptoms (only 1 in 400–600 symptomatic women have OC).^8^ ^9^ Unfortunately, these diagnostic challenges result in nearly 36% of women, subsequently diagnosed with OC, presenting to the GP with symptoms three or more times prior to diagnosis.^10^ Two large prospective studies of symptom-triggered testing for OC suggest that symptom-triggered testing using CA125 is likely to result in referral of a higher proportion of patients with resectable disease.^11^ ^12^

The UK introduced symptom-triggered testing for OC in women with vague symptoms in 2011. National Institute for Health and Care Excellence (NICE) guidelines recommend sequential testing using serum CA125 followed by pelvic ultrasound scan (USS) in women (particularly aged ≥50) presenting to primary care with symptoms such as persistent abdominal distension/‘bloating’, feeling full and/or loss of appetite, pelvic/abdominal pain, increased urinary urgency and/or frequency, unexplained weight loss, fatigue or changes in bowel habit on a persistent or frequent basis.^13^ However, the NICE guidelines do not specify the type of ultrasound abnormalities that should prompt referral. Current tests have limited sensitivity with CA125 being elevated only in 40–50% of women with stage 1 OC in screening and presurgical studies.^14^ ^15^ Once referred to secondary care, women with complex masses considered benign can undergo laparoscopic or conservative management, whereas women with malignancy who undergo surgery by trained gynaecological oncologists have the best outcome.^16^ ^17^ Use of NICE guidelines in practice is extremely variable. A survey of 258 GPs reported that the majority would refer patients on the basis of raised CA125 even if the USS was normal.^18^ A recent audit revealed that the majority of referrals (90%) for suspected OC did not follow the guidelines. Referrals were heterogeneous with regard to which symptoms prompted these, what GPs considered to be abnormal CA125 level or abnormal USS.^19^ Two-thirds of women referred were premenopausal.^19^

Therefore, improved diagnostic tools in primary care and better presurgical triage in secondary care are likely to improve mortality in patients with OC. Optimal diagnostic pathways for premenopausal women with suspected OC/complex ovarian mass and raised CA125 also need to be defined. Ovarian cysts in premenopausal women are extremely common; however, it is important to recognise that about 1000 women under 50 will be diagnosed with OC in the UK annually.^2^

The National Institute of Health Research Health Technology Assessment programme commissioned ROCkeTS—‘Refining Ovarian Cancer Test accuracy Scores’ 13/13/01 (1 October 2014 and 30 September 2018)—which aims to identify, refine and validate tests and clinical risk scores (risk prediction models) that estimate the probability of having OC in postmenopausal and premenopausal women with symptoms, in primary and secondary care. The project comprises four phases/work packages: phase I—systematic reviews of existing risk prediction algorithms and biomarkers to detect OC; phase II—interrogation of data sets from two large OC trials: the screening trial UKCTOCS and the preoperative detection International Ovarian Tumour Analysis (IOTA) studies to derive or refine risk scores with biomarkers from the systematic review; phase III—a prospective study to validate new scores in women newly referred to secondary care with symptoms of OC and phase IV—a model of the diagnostic pathway across primary and secondary care. A cost consequence analysis of testing pathways will be delivered as part of ROCkeTS.

The full ROCkeTS protocol can be found at http://www.birmingham.ac.uk/ROCKETS.

The ROCkeTS project evaluates risk prediction models derived from conventional tests—symptoms, ultrasound variables and commercially available serum blood tests.

Phase III of ROCkeTS is a cross-sectional study aimed at establishing the accuracy of tests and prediction models for the diagnosis of prevalent OC. However, of necessity, the reference standard for a correct diagnosis is follow-up to 12 months, as the study is not restricted to women scheduled for immediate surgery. This is a diagnostic trial (not a screening trial) as women are symptomatic and referred to secondary care.

The phase III prospective study will recruit 2450 premenopausal and postmenopausal symptomatic women over 23 months (recruitment started in June 2015). Patients will be recruited through rapid access clinics, emergency departments or through routine clinic referrals, reflecting the heterogeneity of diagnostic routes for OC in the UK.

Recruited patients consent to complete a validated symptom questionnaire, donate a serum sample and undergo an USS scored as per the IOTA criteria. Standard care pathways are followed beyond this point. Patients are triaged to receive surgery or conservative management based on standard of care model risk of malignancy index (RMI) scores for postmenopausal women, using a threshold of 250 in postmenopausal women or as per local unit practice in premenopausal women. This threshold was selected by the funder as comparator as part of the commissioning brief as this is current standard of care in the UK.^13^ Participants and treating doctors will be blinded to the new biochemical serum test results which will be performed in batches as these are not part of standard clinical care. Outcome data will be collected from histology reports in women undergoing surgery or biopsy and from follow-up over 12 months of those managed conservatively. Symptom scores, serum biomarker tests and ultrasound data will be analysed at the end of follow-up to validate risk prediction models derived in phases I and II.

## ROCkeTS study design

### Aim of the study

- To derive and validate risk prediction models that estimate the probability of having OC for women with symptoms suggestive of OC for postmenopausal and premenopausal women.
- To identify optimal risk thresholds of best models for the diagnosis of OC that can guide patient management.

### Design

The ROCkeTS study is a single-arm prospective cohort diagnostic test accuracy study to evaluate existing and novel risk prediction models for premenopausal and postmenopausal women with symptoms.

A test accuracy study is different from an effectiveness study in that randomisation of participants is not involved. It is designed to generate a comparison of measurements obtained by tests under investigation (index tests) with those obtained from current standard of care tests (comparator test) against a reference standard. In this way, the accuracy of any new ‘index’ tests can be estimated. A reference standard is a test that confirms or refutes the presence or absence of disease beyond reasonable doubt. Therefore, it is sometimes also known as the ‘gold standard’. In this study, as per standards of diagnostic test evaluation, estimates of sensitivity, specificity, c-statistic (area under receiver operating curve (ROC) curve), PPV and negative predictive value (NPV) of tests evaluated and a calibration plot for models evaluated will be presented.

Here, the reference standard will be histology of tissues taken from patients who proceed to surgery or biopsy or follow-up using structured templates at a minimum of 12 months after presentation. The accuracy of the index test will be compared against that of the comparator test, the existing standard risk prediction score RMI. This index is an algorithm combining menopausal status, CA125 and ultrasound features and is standard of care in the UK.^20^ In ROCkeTS, the index test (novel risk prediction models) will be derived in phases I and II and validated in phase III. We will identify biochemical markers, symptom indices and USS as likely components of a novel risk prediction model, as these may be implemented across primary and secondary care. Therefore, we will collect symptom questionnaires, blood and USS data in the study to be analysed and validated at the end of the study.

## Possible components of the new risk prediction model(s)

### Symptoms

Case–control studies demonstrate that symptom questionnaires have good diagnostic accuracy; however, they need to be refined for use by patients in primary care^21^ ^22^ as the duration of symptoms preceding diagnosis is uncertain.^22^ Symptom questionnaires may help triage patients prior to referral and would help standardise symptoms for any prediction model. This is particularly important, given the subjective nature of eliciting symptoms through unstructured clinical history taking and the existing audit evidence that they are interpreted variably by GPs who will only see few cases of OC in their practice. A robust symptom score that can triage referral based on a questionnaire may be very useful.

### Biochemical markers

A number of serum biomarker tests (multiplex testing—OVA1) and multiple-marker-based algorithms (Risk of Ovarian Malignancy Algorithm, ROMA) have been identified in the past decade. Abnormal Human Epididymis 4 (HE4) biomarker levels may improve risk stratification for OC. A recent systematic review reports that HE4 may improve the diagnostic performance of CA125; however, studies showed considerable heterogeneity.^23^

### Ultrasound-based models—IOTA risk prediction models

After publication of an agreement on terms, definitions and measurement methods to describe adnexal masses,^24^ the IOTA collaboration set out multicentre studies on large cohorts of patients presenting with an adnexal mass. The IOTA database has enabled previously developed prediction models to be tested and novel prediction models and rules to characterise ovarian pathology prior to surgery to be developed and validated.^25^ ^26^ In a systematic review with meta-analysis, IOTA algorithms such as the simple rules were identified to be the best presurgical diagnostic tools to characterise adnexal masses, with improved performance over the RMI.^27^ Although the Royal college of Obstetricians and Gynaecologists (RCOG) included the simple rules in their guidance for evaluating ovarian pathology in premenopausal women, IOTA models are not commonly used in National Health Service (NHS) clinical practice.^28^ Recently, the ADNEX (Assessment of Different NEoplasias in the adneXa) model was published. As a multiclass prediction model, it differs from all other models by differentiating between malignant and benign masses and also discriminating between four types of malignant tumours (borderline ovarian tumours, stage I OC, stages II–IV OC and metastatic tumours of other primary origin). ADNEX still needs an extensive external validation but is considered to be promising.^29^

#### Target population

1. Postmenopausal and premenopausal women with symptoms of suspected OC. Symptoms are as defined by NICE guidelines, including but not restricted to persistent or frequent abdominal distension, feeling full (early satiety) and/or loss of appetite, pelvic or abdominal pain, increased urinary urgency and/or frequency and postmenopausal bleeding. Symptoms listed here are not an exhaustive list, and this will be updated from any symptoms identified through phase I systematic reviews of literature.
2. Patients referred with symptoms from GP as suspected OC.

### Comparator

RMI cut-off of 250.^30^

### Source of potential participants

The potential participants are patients who are referred to secondary care as outpatients, either as urgent 2-week or routine referrals, USS clinics, inpatient or emergency presentation.

### Inclusion and exclusion criteria

Multicentre trials across the UK which meet the eligibility criteria are described below.

#### Inclusion criteria

- Newly presenting premenopausal and postmenopausal women with symptoms of suspected OC and either raised CA125, abnormal USS or both. Menopause is defined as >12 months of amenorrhoea.
- Patients aged between 16 and 90 years.
- Patients able to provide informed consent.

NB—Patients with >120 days delay between initial registration IOTA scan and surgery will need a repeat IOTA scan prior to surgery.

#### Exclusion criteria

- USS reveals non-ovarian pathology, for example, fibroids.
- Patients who decline transvaginal scan.
- Previous ovarian malignancy.
- Pregnant patients.
- Patients with previous bilateral oophorectomy.
- Active non-ovarian malignancy—women with a medical history of cancer are eligible only if there are no documented persistent or recurrent diseases and have not received treatment for this in the last 12 months. This exclusion does not apply to patients with premalignant disease, for example, cervical intraepithelial neoplasia or patients receiving tamoxifen/other drugs to prevent breast cancer recurrence.

**Figure 1:**
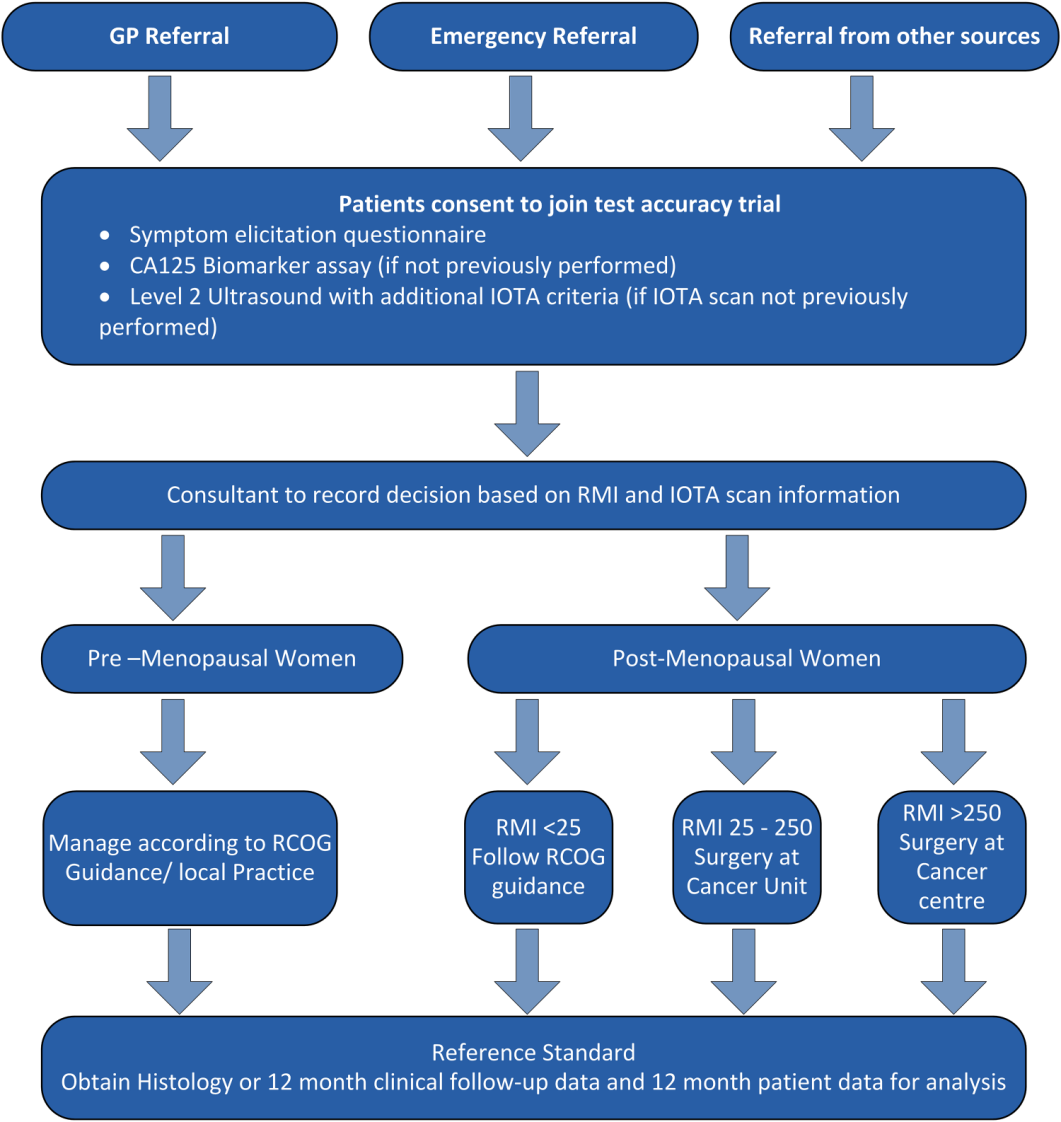
Flow chart. GP, general practitioner; IOTA, International Ovarian Tumour Analysis; RCOG, Royal College of Obstetricians and Gynaecologists; RMI, risk of malignancy index.

See figure 1 for participant flow through the trial.

## Study procedures and tests

### The index tests

There are three index tests that will be performed in the prospective single-arm test accuracy study.

1. Participants entering the study will complete a symptom elicitation questionnaire and anxiety questionnaire (STA6) and impact of event score.
2. Ultrasonographers will record the USS variables and score the USS using IOTA simple rules and ADNEX models.^29^ ^31^ ^32^ For most women in the trial, this will only mean some additional data being collected during their USS appointment. For a small number of women, this may mean an additional USS after consent.
3. Participants will have an additional blood sample taken at baseline for biomarker assessment at the end of the study. Serum will be banked for testing at a later point. Treating doctors will be blinded to any new biomarker assessments performed as part of this trial. Details of blood sample collection will be provided in a laboratory manual.

Apart from the study tests, all other aspects of participant management are entirely at the discretion of the local doctors and as per the RCOG guidelines for the management of these participants.^28^ ^30^ Treating clinicians will be asked to record their treatment recommendations as per standard care and after any additional USS information to assess the impact of this test on care pathways.

### Quality assurance of index tests

The performance of USS is subjective and operator dependent. Therefore, sonographers/doctors carrying out USS will undergo a face-to-face IOTA training course provided as part of their participation in the ROCkeTS trial. Sites will commit to undergoing quality assurance during the ROCkeTS trial. Online USS training materials will be developed during this project for future use by the UK NHS. Quality assurance of testing will begin with a clearly documented staff training programme. A register of staff who have been trained and had their competence assessed will be maintained, and only staff whose names appear on this list will be permitted to undertake scans within the ROCkeTS prospective trial. Staff will also receive a site visit and assessment of their competence. Competence will be assessed by those authorised by the IOTA team.

### Reference standard/follow-up schedule

Reference standard for the study will be histology of tissue taken at surgery or biopsy in women who are managed surgically following study enrolment. Histology data collected will include information on whether the tumour was considered invasive cancer or borderline. The outcome of participants referred for suspected OC who do not undergo surgery will be assessed by a follow-up visit at 12 months, by a telephone call or a questionnaire from the research nurse at 12 months, as per the local investigators' discretion and clinical assessment. Well-being will be ascertained at this follow-up. A structured template will be used. Women will also be asked to give permission for data to be linked with cross-checked against national cancer registry data so that we are able to identify the false-negative rate for any diagnostic tests.

### Sample size

The diagnostic accuracy from models will be compared to the current tests recommended by NICE for primary and secondary care. The sample size has a 90% power to detect with 5% significance difference in accuracy between the existing test (RMI threshold 250) and the new model. Owing to the expected difference in performance in premenopausal and postmenopausal women, separate sample sizes have been calculated. Sample sizes are calculated assuming independence of test errors and interim analyses will confirm parameter assumptions.

#### For postmenopausal women

The performance of RMI at threshold 250 is assumed to be 70% sensitive and 90% specific.^30^ One thousand and four hundred participants will be required to detect an increase in sensitivity of 10% (to 80%) and in specificity of 5% (to 95%). Based on a prevalence of 30% of OC in referred women (local audit figures), with sensitivity and specificity of RMI assumed to be 70% and 90%, a sample size of 1333 provides 90% power to detect an increase of sensitivity to 80% and specificity to 95% in paired data (conservatively assuming independence of test errors). Allowing for a loss to follow-up of up to 5%, this gives a final sample size of 1400.

#### For premenopausal women

The performance of RMI is assumed to have a sensitivity of 72% and a specificity of 46% (local audit figures). The trial is powered to detect an increase in sensitivity of 10% (to 82%) and in specificity of 10% (to 56%). Prevalence of OC in premenopausal women referred to secondary care is about 10%.^19^ A sample size of 1000 will provide 100 OC events in which to build new models combining symptom and test data (adequate events to model 10 predictor variables) and will provide 90% power to detect an increase in specificity of 8% (from 46% for RMI to 54%). With a predicted loss to follow-up of up to 5%, the final sample size required is 1050 women.

### Study duration

We anticipate recruitment of 2450 participants within 23 months, with a minimum of 12 months follow-up from the last participant entering the study.

### Data collection

All information will be collected on standard proformas (case report forms; CRF) and identified by study number. Information will be collated on paper forms and then either copied and sent to the coordinating centre for input or entered directly into the study database via a web interface. A data set including age, ethnicity, parity, GP details and significant medical/surgical history will be collected. We aim to use the NHS number as the primary identifier when linking to national registries and to track individuals throughout the NHS. Additional data on outcomes such as cancer or non-cancereous conditions will be collected at follow-up.

Data will be collected on relevant medical, obstetric and gynaecological, surgical history, emotional impact as well as information on the symptoms that prompted GP referral or investigation. USS information will be collected. Data on the reference diagnosis will be obtained from the histopathology report and a structured template to assess well-being for participants who do not undergo surgery will be collected directly from the participants. Importantly, outcomes collected will include all conditions/diagnoses in women with non-specific symptoms.

### Measures and costs

Validation of a risk prediction model and tests for estimating the probability of OC in women with suspected OC, key outcome measures include the accuracy of the tests and models in terms of their discrimination ability (eg, sensitivity, specificity) and calibration (observed vs predicted probabilities), and the identification of thresholds to guide patient management decisions.

Trial data collection will be undertaken prospectively for all participants in order to inform the costs for each pathway.

### Analysis plan—test accuracy

We will report estimates of sensitivity, specificity, c-statistic (area under ROC curve), PPV and NPV of tests evaluated and a calibration plot for models evaluated. Also, in terms of our new model derived from phase II of the wider ROCkeTS project, its improvement over existing models will be summarised by comparing the c-statistic and the calibration. We will also summarise the net-reclassification index for each new predictor that existing models omitted.

The risk prediction models derived in phases I and II of the ROCkeTS project will each produce a predicted risk of OC by 12 months for all the individuals in our study. Therefore, we will compare the observed outcome at 12 months with this predicted risk. The calibration (in terms of calibration slope) and discrimination (eg, c-statistic) will be evaluated for the models derived and identified in phase I and phase II, and their performance will be compared to the existing RMI model. The calibration will be shown visually by grouping women into deciles ordered by predicted risk and considering the agreement between the mean predicted risk and the observed events in each decile. The aim was to use predefined models on the phase III data, so the bulk of phase III analysis will be external validation of predefined models. Where the data set is used to derive a new model, optimism will be reduced using shrinkage methods through internal validation.

### Generalisability of results to primary care

We appreciate that there may be recalibration required for any models validated within ROCkeTS in the primary care setting. However, we stress that to conduct a study that recruited women in primary care to validate diagnostic models would need to be extremely large and prohibitively expensive. Furthermore, our work and others have shown that GPs are likely to refer on the basis of a raised CA125 or abnormal scan rather than follow the NICE suggested referral pathway which is based on sequential CA125 and USS and referral only if both are abnormal.^18^ ^19^ Thus, we believe that the population of patients referred through rapid access clinics with symptoms will be more heterogeneous than anticipated from the NICE guidelines and therefore maybe more applicable to a primary care population.

### Analysis plan—cost consequence analysis

Resource usage for each of the diagnostic tests will be broken down and displayed along with their unit costs alongside the outcomes for each pathway. The resource usage will include the types of tests administered, the number of inpatient and outpatient consultations, and any operative procedures undertaken. This approach will help to show which are the major cost drivers for each of the diagnostic pathways and will be collected as part of the clinical CRF.

### Study conduct

The conduct of the study will be in accordance with the Research Governance Framework for Health and Social Care and/or the Research Governance Framework for Health and Community Care. The participant's written informed consent to participate in the trial will be obtained before any trial procedures or questionnaires are completed. The women's GP will be notified of her participation in the study with her consent (GP letter).

Participants will be free to withdraw from the study at any time without any effect on their standard of care; data and samples provided up to the point a participant withdraws will be retained unless the participant expressly requests their removal. This is because analysis will be based on all recruited participants and per protocol.
